# A Batch Rival Penalized Expectation-Maximization Algorithm for Gaussian Mixture Clustering with Automatic Model Selection

**DOI:** 10.1155/2012/425730

**Published:** 2012-01-30

**Authors:** Jiechang Wen, Dan Zhang, Yiu-ming Cheung, Hailin Liu, Xinge You

**Affiliations:** ^1^Faculty of Applied Mathematics, Guangdong University of Technology, Guangzhou 510520, China; ^2^Department of Computer Science, Hong Kong Baptist University, Kowloon, Hong Kong; ^3^Department of Electronics and Information Engineering, Huazhong University of Science &Technology, Wuhan, China

## Abstract

Within the learning framework of maximum weighted likelihood (MWL) proposed by Cheung, 2004 and 2005, this paper will develop a batch Rival Penalized Expectation-Maximization (RPEM) algorithm for density mixture clustering provided that all observations are available before the learning process. Compared to the adaptive RPEM algorithm in Cheung, 2004 and 2005, this batch RPEM need not assign the learning rate analogous to the Expectation-Maximization (EM) algorithm (Dempster et al., 1977), but still preserves the capability of automatic model selection. Further, the convergence speed of this batch RPEM is faster than the EM and the adaptive RPEM in general. The experiments show the superior performance of the proposed algorithm on the synthetic data and color image segmentation.

## 1. Introduction

As a typical statistical technique, clustering analysis has been widely applied to a variety of scientific areas such as data mining [1], vector quantization [2, 3], image processing [4–7], and so forth. In particular, clustering analysis provides a useful tool to solve the several computer vision problems, for example, multithresholding of gray level images, analysis of the Hough space, and range image segmentation, formulated in the feature space paradigm [8]. In general, one kind of clustering analysis can be formulated as a density mixture modeling, in which each mixture component represents the density distribution of a data cluster. Subsequently, the task of clustering analysis is to identify the dense regions of the input (also called *observation* interchangeably) densities in a mixture. Such a clustering is therefore called a density mixture clustering.

In general, the Expectation-Maximum (EM) algorithm [9, 10] has provided a general solution for the parameter estimation of a density mixture model. Nevertheless, it needs to preassign an appropriate number of density components, that is, the number of clusters. Roughly, the mixture may overfit the data if too many components are utilized, whereas a mixture with too few components may not be flexible enough to approximate the true underlying model. Subsequently, the EM almost always leads to a poor estimate result if the number of components is misspecified. Unfortunately, from the practical viewpoint, it is hard or even impossible to know the exact cluster number in advance. In the literature, one promising way is to develop a clustering algorithm that is able to perform a correct clustering without preassigning the exact number of clusters. Such algorithms include the RPCL algorithm [11] and its improved version, namely, RPCCL [12]. More recently, Cheung [13, 14] has proposed a general learning framework, namely, Maximum Weighted Likelihood (MWL), through which an adaptive Rival Penalized EM (RPEM) algorithm has been proposed for density mixture clustering. The RPEM learns the density parameters by making mixture component compete each other at each time step. Not only are the associated parameters of the winning density component updated to adapt to an input, but also all rivals' parameters are penalized with the strength proportional to the corresponding posterior density probabilities. Therefore, this intrinsic rival penalization mechanism enables the RPEM to automatically select an appropriate number of densities by gradually fading out the redundant densities from a density mixture. Furthermore, a simplified version of RPEM has included RPCL and RPCCL as its special cases with some new extensions.

In the papers [13, 14], the RPEM algorithm learns the parameters via a stochastic gradient ascending method; that is, we update the parameters immediately and adaptively once the current observation is available. In general, the adaptiveness of the RPEM makes it more applicable to the environment changed over time. Nevertheless, the convergence speed of the RPEM relies on the value of learning rate. Often, by a rule of thumb, we arbitrarily set the learning rate at a small positive constant. If the value of learning rate is assigned too small, the algorithm will converge at a very slow speed. On the contrary, if it is too large, the algorithm may even oscillate. In general, it is a nontrivial task to assign an appropriate value to the learning rate, although we can pay extra efforts to make the learning rate dynamically change over time, for example, see [15].

In this paper, we further study the MWL learning framework and develop a batch RPEM algorithm accordingly provided that all observations are available before the learning process. Compared to the adaptive RPEM, this batch one need not assign the learning rate analogous to the EM, but still preserves the capability of automatic model selection. Further, the convergence speed of this batch RPEM is faster than the EM and the adaptive RPEM in general. The experiments have shown the superior performance of the proposed algorithm on the synthetic data and color image segmentation.

The remainder of this paper is organized as follows.  Section 2 reviews the MWL learning framework. In Section 3, we present the batch RPEM algorithm in detail, in which the weights involve a coefficient *ɛ*. We will therefore further explore the assignment of *ɛ* in Section 4.  Section 5 shows the detailed experiment results. Finally, we draw a conclusion in Section 6.

## 2. Overview of Maximum Weighted Likelihood (MWL) Learning Framework

Suppose that an input **x** ∈ *ℜ* ^*d*^ comes from the following density mixture model:

(1) $$\begin{array}{cc} P\left(x\;|\;\Theta \right)=\overset{k}{\underset{j=1}{\sum }}\alpha_{j}p\left(x\;|\;\theta_{j}\right),\;\overset{k}{\underset{j=1}{\sum }}\alpha_{j}=1, & \\ \alpha_{j}>0,\;\forall 1\leq j\leq k, & \end{array}$$

where Θ is the parameter set of {*α*_*j*_, ***θ***_*j*_}_*j*=1_^*k*^. Furthermore, *k* is the number of components, *α*_*j*_ is the mixture proportion of the *j*th component, and *p*(**x** | ***θ***_*j*_) is a multivariate probability density function (pdf) of **x** parameterized by ***θ***_*j*_. As long as we know the value of Θ, an input **x** can be classified into a certain cluster via its posterior probability:

(2) $$\begin{array}{c} \begin{array}{cc} h\left(j\;|\;x,\Theta \right) & =\frac{\alpha_{j}p\left(x\;|\;\theta_{j}\right)}{P\left(x\;|\;\Theta \right)} \end{array} \end{array}$$

using the winner-take-all rule, that is, assigning an input **x** to Cluster *c* if *c* = argmax⁡_*j*_ *h*(*j* | **x**, Θ) or taking its soft version which assigns **x** to Cluster *j* with the probability *h*(*j* | **x**, Θ).  Therefore, how to estimate the parameter set Θ, particularly without knowing the correct value of *k* in advance, is a key issue in density mixture clustering.

In the MWL learning framework [13, 14], the parameter set Θ is learned via maximizing the following Weighted Likelihood (WL) cost function:

(3) $$\begin{array}{c} l\left(\Theta \right)=\omega \left(\Theta ;x\right)+\nu \left(\Theta ;x\right) \end{array}$$

with

(4) $$\begin{array}{c} \omega \left(\Theta ;x\right)=\int \overset{k}{\underset{j=1}{\sum }}g\left(j\;|\;x,\Theta \right)\ln\left[\alpha_{j}p\left(x\;|\;\theta_{j}\right)\right]dF\left(x\right),\\ \nu \left(\Theta ;x\right)=-\int \overset{k}{\underset{j=1}{\sum }}g\left(j\;|\;x,\Theta \right)\ln h\left(\begin{array}{c} j\;|\;x,\Theta \end{array}\right)dF\left(x\right), \end{array}$$

where *g*(*j* | **x**, Θ)'s are the designable weights satisfying the two conditions:

(1) ∑_*j*=1_^*k*^ *g*(*j* | **x**, Θ) = 1,
(2) for all  *j*,  *g*(*j* | **x**, Θ) = 0  if  *h*(*j* | **x**, Θ) = 0.

Suppose that a set of *N* i.i.d. observations, denoted as **X** = {**x**_1_, **x**_2_,…, **x**_*N*_}, comes from the density mixture model in (1). The empirical WL function of (3), written as *Q*(Θ; **X**), can be given as

(5) $$\begin{array}{c} Q\left(\Theta ;X\right)=\omega \left(\Theta ;X\right)+\nu \left(\Theta ;X\right) \end{array}$$

with

(6) $$\begin{array}{c} \omega \left(\Theta ;X\right)=\frac{1}{N}\overset{N}{\underset{t=1}{\sum }}\overset{k}{\underset{\;\;j=1}{\sum }}g\left(\begin{array}{c} j\;|\;x_{t},\Theta \end{array}\right)\ln\left[\alpha_{j}p\left(x_{t}\;|\;\theta_{j}\right)\right],\\ \nu \left(\Theta ;X\right)=-\frac{1}{N}\overset{N}{\underset{t=1}{\sum }}\overset{k}{\underset{\;\;j=1}{\sum }}g\left(j\;|\;x_{t},\Theta \right)\ln h\left(j\;|\;x_{t},\Theta \right). \end{array}$$

Moreover, the weights *g*(*j* | **x**_*t*_, Θ)'s have been generally designed as [13, 14]

(7) $$\begin{array}{c} g\left(j\;|\;x_{t},\Theta \right)=\left(1+{ɛ}_{t}\right)I\left(j\;|\;x_{t}\text{,}\Theta \right)-{ɛ}_{t}h\left(j\;|\;x_{t},\Theta \right), \end{array}$$

where *ɛ*_*t*_ is a coefficient varying with the time step *t* in general. Please note that *g*(*j* | **x**_*t*_, Θ)'s in (7) can be negative as well as positive. For simplicity, we hereinafter set *ɛ*_*t*_ as a constant, denoted as *ɛ*. Moreover, *I*(*j* | **x**_*t*_,  Θ) is an indicator function with

(8) $$\begin{array}{c} I\left(j\;|\;x_{t},\Theta \right)=\{\begin{array}{c} 1,\;\text{if}\;\;j=c=\arg\underset{1\leq j\leq k}{\max}h\left(j\;|\;x_{t},\Theta \right),\\ 0,\;\text{otherwise.} \end{array} \end{array}$$

Subsequently, under a specific weight design, the papers [13, 14] have presented the adaptive RPEM to learn Θ via maximizing the WL function of (5) using a stochastic gradient ascent method, which is able to fade out the redundant densities gradually from a density mixture. Consequently, it can automatically select an appropriate number of density components in density mixture clustering. Interested readers may refer to the paper [14] for more details. We summarize the main steps of the adaptive RPEM in Algorithm 1. Although the experiments have shown the superior performance of the adaptive RPEM on automatic model selection, its convergence speed, however, relies on the value of learning rate. Under the circumstances, we will present a batch version without the learning rate in the next section. 

## 3. Batch RPEM Algorithm

To estimate the parameter set within the MWL framework, we have to maximize the empirical WL function *Q*(Θ; **X**) in (5). In general, we update the parameters via maximizing the first term of (5), that is, *ω*(Θ; **X**), by fixing the second term *ν*(Θ; **X**). Subsequently, we need to solve the following nonlinear optimization problem:

(9) $$\begin{array}{c} \tilde{\Theta }=\arg\underset{\Theta }{\max}\left\{\omega \left(\Theta ;X\right)\right\} \end{array}$$

subject to the constraints as shown in (1). We adopt the Lagrange method analogous to the EM by introducing a Lagrange multiplier *λ* into the Lagrange function. Subsequently, we have

(10) $$\begin{array}{c} ℒ\left(\Theta ,\lambda \right)=\omega \left(\Theta ;X\right)+\lambda \left(\overset{k}{\underset{j=1}{\sum }}\alpha_{j}-1\right). \end{array}$$

In this paper, we concentrate on the Gaussian mixture model only, that is, each component *p*(**x** | ***θ***_*j*_) in (1) is a Gaussian density. We then have

(11) $$\begin{array}{cc} & p\left(j\;|\;x_{t},\theta_{j}\right)\\ & \;=\begin{array}{c} {\left(2\pi \right)}^{-d/2}{\left|C_{j}\right|}^{-1/2}\exp\left[-\frac{1}{2}{\left(x_{t}-\mu_{j}\right)}^{T}C_{j}^{-1}\left(x_{t}-\mu_{j}\right)\right], \end{array} \end{array}$$

where ***θ***_*j*_ = (***μ***_*j*_, **C**_*j*_), ***μ***_*j*_ and **C**_*j*_ are the mean (also called *seed points* interchangeably) and the covariance of the *j*th density, respectively.

Through optimizing (10), we then finally obtain the batch RPEM algorithm as shown in Algorithm 2. If a covariance matrix **C**_*j*_^(*n*+1)^ is singular, then it indicates that the corresponding *j*th density component is degenerated and can be simply discarded without being learned any more in the subsequent iterations. In this case, we have to normalize those remaining *α*_*r*_^(*n*+1)^'s (*r* ≠ *j*) so that their sum is always kept to be 1.

In the above batch RPEM, its capability of automatic model selection is controlled by the weight functions *g*(*j* | **x**_*t*_, ***θ***)'s, which further rely on the parameter *ɛ* as shown in (7). Subsequently, a new question is arisen: how to assign an appropriate value of *ɛ*? The next section will answer this question.

## 4. How to Assign Parameter  *ɛ*?

To deal with how to assign an appropriate value of *ɛ*, we rewrite (7) as the following form:

(12) $$\begin{array}{cc} & g\left(j\;|\;x_{t},\Theta \right)\\ & =\{\begin{array}{cc} \left(1+ɛ\right)I\left(c\;|\;x_{t},\Theta \right)-ɛh\left(c\;|\;x_{t},\Theta \right), & \text{if}\;j=c,\\ -ɛh\left(j\;|\;x_{t}\right), & \text{otherwise} \end{array}\\ & =\{\begin{array}{cc} h\left(c\;|\;x_{t},\Theta \right)+\left(1+ɛ\right)\left(1-h\left(c\;|\;x_{t},\Theta \right)\right), & \text{if}\;j=c,\\ h\left(j\;|\;x_{t},\Theta \right)-\left(1+ɛ\right)h\left(j\;|\;x_{t},\Theta \right), & \text{otherwise}. \end{array} \end{array}$$

Intuitively, the term (1 + *ɛ*)(1 − *h*(*c* | **x**_*t*_, Θ)) can be regarded as the award received by the winning density component (i.e., the *c*th density with *I*(*c* | **x**_*t*_, Θ) = 1), and meanwhile the term −(1 + *ɛ*)*h*(*j* | **x**_*t*_, Θ) is the penalty of the rival components (i.e., those densities with *I*(*j* | **x**_*t*_, Θ) = 0). In general, it is expected that the award is positive and the penalty is negative. That is, *ɛ* should be greater than −1. Otherwise, as *ɛ* < −1, we will meet an awkward situation; that is, the amount of award is negative and the penalty one becomes positive. This implies that we will penalize the winner and award the rivals, which evidently violates our expectations. Furthermore, as *ɛ* = −1, both of the award and penalty amount become zero. In this special case, the batch RPEM is actually degenerated into the EM without the property of automatic model selection. As a result, *ɛ* is required to be greater than −1. In addition, *ɛ* in the batch RPEM should be a negative value. Otherwise, the weights of the rival components *g*(*j* | **x**_*t*_, Θ) = −*ɛh*(*j* | **x**_*t*_)'s become negative, resulting in some *α*_*j*_'s to be negative finally. Hence, an appropriate selection of *ɛ* in the batch RPEM would be a negative value and greater than −1. That is, *ɛ* should be fallen into the range of (−1,0).

**Figure 1:**
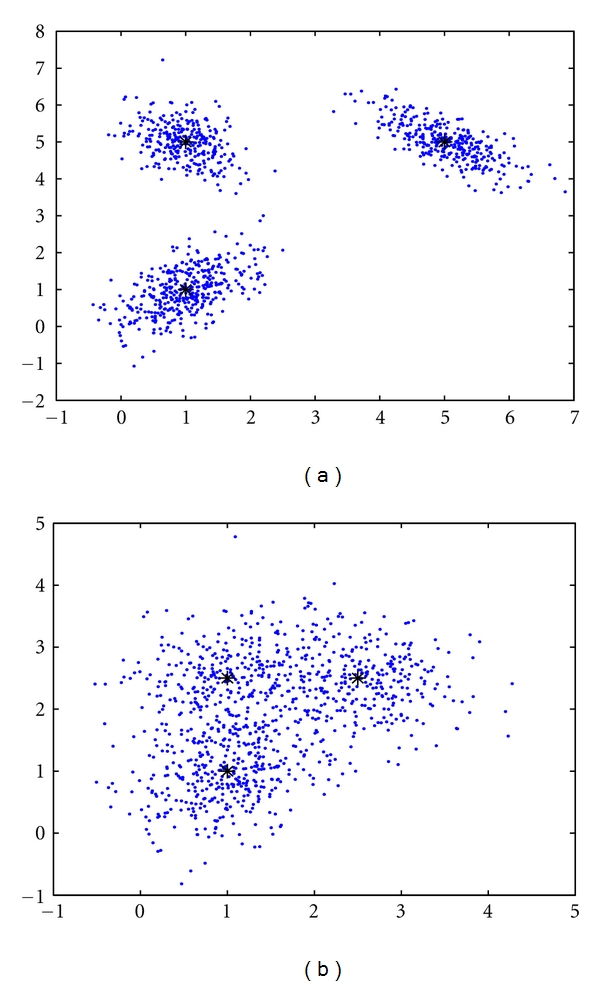
(a) Synthetic data set 1 with the well-separated clusters, and (b) synthetic data set 2 with the clusters overlapped considerably.

Furthermore, our empirical studies have found that a smaller *ɛ* will lead the batch RPEM algorithm to a more robust performance, especially when the value of *k* is large and the data are overlapped considerably. In other words, the algorithm has a poor capability of automatic model selection if *ɛ* is close to zero. To illustrate this scenario, we have utilized two synthetic data sets that are generated from the two bivariate three-Gaussian mixtures individually as shown in Figures 1(a) and 1(b), where each data set consists of 1,000 observations with the true mixture proportions: *α*_1_* = 0.4, *α*_2_* = 0.3, and *α*_3_* = 0.3. Also, the true ***μ***_j_*'s and **C**_j_*'s of data set 1 in Figure 1(a) are

(13) $$\begin{array}{c} \mu_{1}^{*}\begin{array}{c} =\left(\begin{array}{c} 1.0\\ 1.0 \end{array}\right),\;{\;\;\mu }_{2}^{*}=\left(\begin{array}{c} 1.0\\ 5.0 \end{array}\right),\;\;\; \end{array}\mu_{3}^{*}=\left(\begin{array}{c} 5.0\\ 5.0 \end{array}\right),\\ \begin{array}{c} C_{1}^{*}=\left(\begin{array}{cc} 0.3 & 0.2\\ 0.2 & 0.4 \end{array}\right),\;\;C_{2}^{*}=\left(\begin{array}{cc} 0.2 & -0.1\\ -0.1 & 0.3 \end{array}\right),\;\\ C_{3}^{*}=\left(\begin{array}{cc} 0.30 & -0.20\\ -0.20 & 0.25 \end{array}\right), \end{array}\; \end{array}$$

while the true parameters of data set 2 in Figure 1(b) are

(14) $$\begin{array}{c} \begin{array}{c} \begin{array}{c} \mu_{1}^{*}=\left(\begin{array}{c} 1.0\\ 1.0 \end{array}\right),\;\;\;\mu_{2}^{*}=\left(\begin{array}{c} 1.0\\ 2.5 \end{array}\right),\; \end{array}\;\;\mu_{3}^{*}=\left(\begin{array}{c} 2.5\\ 2.5 \end{array}\right),\\ C_{1}^{*}=\left(\begin{array}{cc} 0.3 & 0.1\\ 0.1 & 0.4 \end{array}\right),\;\;C_{2}^{*}=\left(\begin{array}{cc} 0.3 & 0.0\\ 0.0 & 0.3 \end{array}\right),\;\;\\ C_{3}^{*}=\left(\begin{array}{cc} 0.30 & -0.05\\ -0.05 & 0.25 \end{array}\right). \end{array}\; \end{array}$$

It can be seen that the clusters in data set 1 are well separated, whereas the clusters in data set 2 are overlapped considerably.

**Figure 2:**
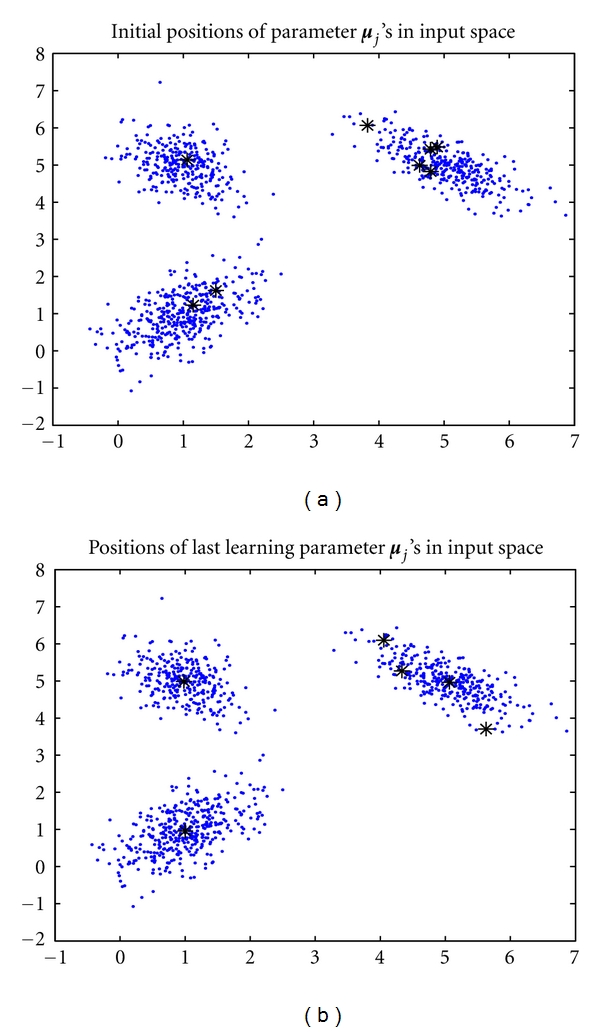
The performance of the batch RPEM as k* = 3, *k* = 8, and *ɛ* = −0.8: (a) initial positions of seed points; (b) converged positions of seed points.

For each data set, we conducted the three experiments by setting *k* = 3, *k* = 8, and *k* = 20, respectively. Also, all *α*_*j*_'s and **C**_*j*_'s were initialized at 1/*k* and the identity matrix, respectively. During the learning process, we discarded those densities whose covariance matrices **C**_*j*_'s were singular.  Table 1 shows the performance of the batch RPEM over the parameter *ɛ*. We found that, as *k* = 3 and *k* = 8, all *ɛ*'s we have tried from −0.9 to −0.1 lead to the good performance of the algorithm when using the data set 1. For example, as *k* = 8 and *ɛ* = −0.8, we randomly initialized the eight seed points in the input space as shown in Figure 2(a). After all the parameters were converged, 2 out of 8 density components had been discarded and the mixture proportions of the remaining components were converged to *α*_1_ = 0.2960, *α*_2_ = 0.0036, *α*_3_ = 0.2900, *α*_4_ = 0.0058, *α*_5_ = 0.0136, and *α*_6_ = 0.3910. It can be seen that the three principal mixing proportions, *α*_1_, *α*_3_, and *α*_6_, have well estimated the true ones, while the other proportions were inclined to zero. The corresponding three ***μ***_*j*_'s and **C**_*j*_'s were

(15) $$\begin{array}{c} \begin{array}{c} \mu_{1}=\left(\begin{array}{c} 5.06\\ 4.96 \end{array}\right),\;\;\;\mu_{3}=\left(\begin{array}{c} 0.98\\ 4.98 \end{array}\right),\;\;\mu_{6}=\left(\begin{array}{c} 1.00\\ 0.96 \end{array}\right), \end{array}\\ \begin{array}{c} C_{1}=\left(\begin{array}{cc} 0.29 & -0.17\\ -0.17 & 0.22 \end{array}\right),\;\;C_{3}=\left(\begin{array}{cc} 0.18 & -0.08\\ -0.08 & 0.25 \end{array}\right),\;\\ C_{6}=\left(\begin{array}{cc} 0.29 & 0.19\\ 0.19 & 0.39 \end{array}\right). \end{array}\; \end{array}$$

As shown in Figure 2(b), the three ***μ***_*j*_'s have successfully stabilized at the corresponding cluster centers, meanwhile the other three redundant seed points have been pushed away and stably located at the boundary of the clusters. That is, the redundant densities have been fade out through the learning, thus the batch RPEM can select the model automatically as well as the adaptive version.

**Figure 3:**
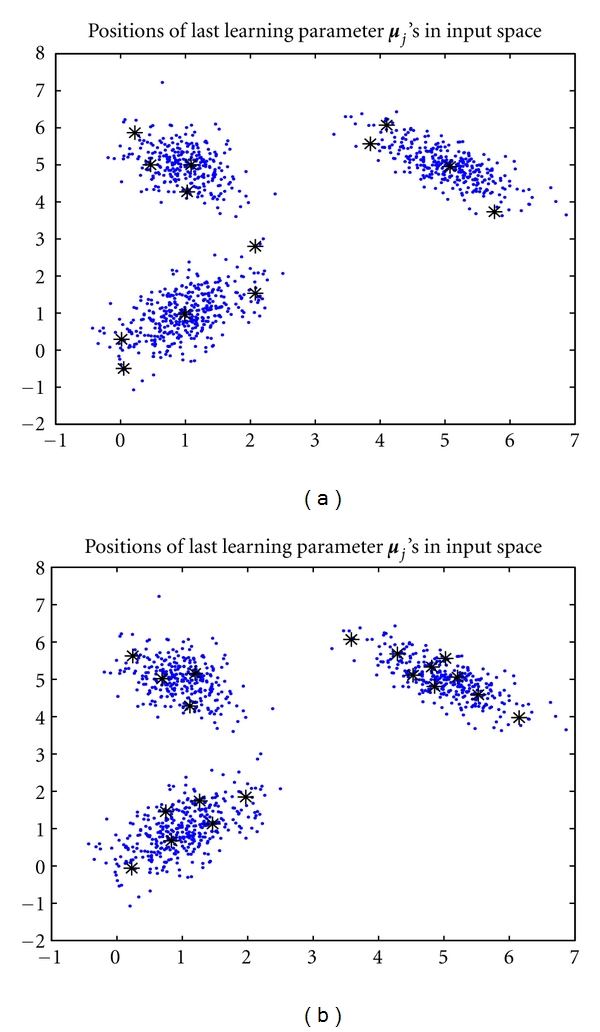
The converged positions of the seed points as k* = 3 and *k* = 20: (a) *ɛ* = −0.9, (b) *ɛ* = −0.1.

Nevertheless, when *k* is set at a large value, for example, say *k* = 20, it is found that the proposed algorithm could not fade out the redundant density components from a mixture if *ɛ* is close to 0. Instead, we should set *ɛ* at a value close to −1. For example, as k* = 3, *k* = 20, and *ɛ* = −0.9, we ran the proposed algorithm. It was found that 13 of 20 seed points were maintained by discarding those whose covariance matrices **C**_*j*_'s were singular. The mixture proportions of the remaining components were converged to *α*_1_ = 0.0421, *α*_2_ = 0.0169, *α*_3_ = 0.0051, *α*_4_ = 0.2349, *α*_5_ = 0.0036, *α*_6_ = 0.0149, *α*_7_ = 0.3444, *α*_8_ = 0.0049, *α*_9_ = 0.0210, *α*_10_ = 0.0029, *α*_11_ = 0.0057, *α*_12_ = 0.2944, and *α*_13_ = 0.0091. The three principal mixing proportions, *α*_4_, *α*_7_, and *α*_12_, have also well estimated the true ones while the other proportions were tended to zero. Furthermore, the corresponding ***μ***_*j*_'s were ***μ***_1_ = [1.0872,4.9986]^*T*^, ***μ***_2_ = [0.9897,0.9640]^*T*^, and ***μ***_3_ = [5.0754,4.9552]^*T*^. As shown in Figure 3(a), the learned ***μ***_*j*_'s are correctly allocated at the center of the three clusters and the other redundant seed points were driven away to the boundaries of clusters. Hence, the batch algorithm performed a good model selection by assigning *ɛ* = −0.9. In contrast, if we assign *ɛ* to some value close to zero, the algorithm will lead to a poor model selection. We take *ɛ* = −0.1 for instance. The mixture proportions of the remaining 19 out of 20 components were converged to *α*_1_ = 0.0461, *α*_2_ = 0.0121, *α*_3_ = 0.0439, *α*_4_ = 0.1404, *α*_5_ = 0.0070, *α*_6_ = 0.0258, *α*_7_ = 0.0178, *α*_8_ = 0.0348, *α*_9_ = 0.0659, *α*_10_ = 0.0513, *α*_11_ = 0.0493, *α*_12_ = 0.0352, *α*_13_ = 0.0362, *α*_14_ = 0.0528, *α*_15_ = 0.0587, *α*_16_ = 0.0171, *α*_17_ = 0.1916, *α*_18_ = 0.0882, and *α*_19_ = 0.0260. It can be seen that none of *α*_*j*_'s tends to zero. As shown in Figure 3(b), all the converged positions have a bias from the cluster centers. In other words, the algorithm has a poor performance as *ɛ* get close to zero. Hence, if *k* is large, it would be better to choose a relative smaller value of *ɛ* between −1 and 0.

**Figure 4:**
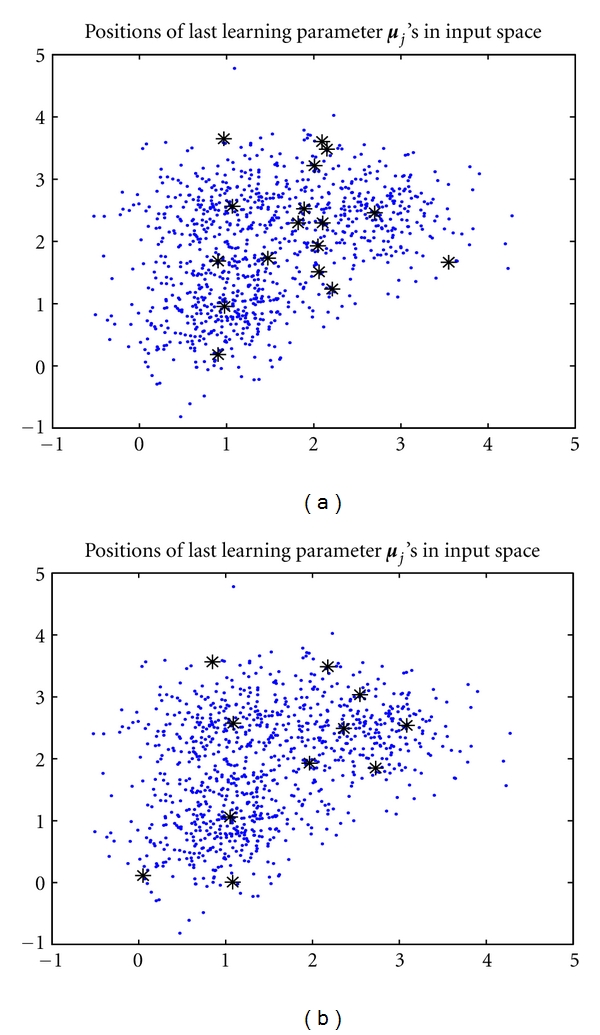
The converged positions of the seed points learned via the batch RPEM as k* = 3 and *k* = 20: (a) *ɛ* = −0.9; (b) *ɛ* = −0.2.

In addition, we also investigated the assignment of *ɛ* on data set 2, where the data are considerably overlapped. We take k* = 3, *k* = 20, and *ɛ* = −0.9 for instance. The converged positions of the seed points are shown in Figure 4(a), where the learned positions converged to the cluster centers while driving the redundant seed points to the boundaries of the clusters. That is, the proposed batch algorithm can work quite well as *ɛ* = −0.9. Also, we let k* = 3, *k* = 20, and *ɛ* = −0.2 to run the algorithm again for comparison. As shown in Figure 4(b), the converged positions of the seed points have a bias from the cluster centers. This implies that the values of *ɛ* that are close to zero cannot work well in this case. More examples can be found in Table 1. It can be seen that the feasible region of *ɛ* is shrunk over the overlap level of the data. For example, the appropriate values of *ɛ* are in the range of [−0.9, −0.6] only when using the date set 2 with k* = 3 and *k* = 3 or 8, respectively. In contrast, *ɛ* is feasible in the full range of [−0.9, −0.1] where we have tried so far as data set 1 is used. Hence, by a rule of thumb, we should choose an appropriate value of *ɛ* close to −1. Nevertheless, we have also noted that it is not a good choice if *ɛ* is too close to −1. In fact, the proposed algorithm will gradually degenerate to the EM as *ɛ* tends to −1; that is, the capability of the proposed algorithm on model selection will be reduced as *ɛ* tends to −1. Hence, to sum up, empirical studies have found that [−0.9, −0.8] is an appropriate feasible region of *ɛ*. In the next section, we therefore arbitrarily set *ɛ* at −0.8. 

## 5. Experimental Results 

To evaluate the performance of the batch RPEM algorithm, we have conducted the following three experiments.

### 5.1. Experiment  1: Batch RPEM on Synthetic Data with *K* = K*

This experiment was to evaluate the convergence speed of the batch RPEM. We utilized 1,000 data points from a mixture of three bivariate Gaussian densities with the true parameters as follows:

(16) $$\begin{array}{c} \begin{array}{c} \alpha_{1}^{*}=0.3,\;\;\;\alpha_{2}^{*}=0.4,\;{\;\;\alpha }_{3}^{*}=0.3, \end{array}\\ \begin{array}{c} \mu_{1}^{*}={\left[1.0,1.0\right]}^{T},\;{\;\mu }_{2}^{*}={\left[1.0,2.5\right]}^{T},\;\mu_{3}^{*}={\left[2.5,2.5\right]}^{T},\; \end{array}\\ \begin{array}{c} C_{1}^{*}=\left(\begin{array}{cc} 0.20 & 0.05\\ 0.05 & 0.30 \end{array}\right),\;{\;\;C}_{2}^{*}=\left(\begin{array}{cc} 0.2 & 0.0\\ 0.0 & 0.2 \end{array}\right),\;\\ C_{3}^{*}=\left(\begin{array}{cc} 0.2 & -0.1\\ -0.1 & 0.2 \end{array}\right). \end{array} \end{array}$$

**Figure 5:**
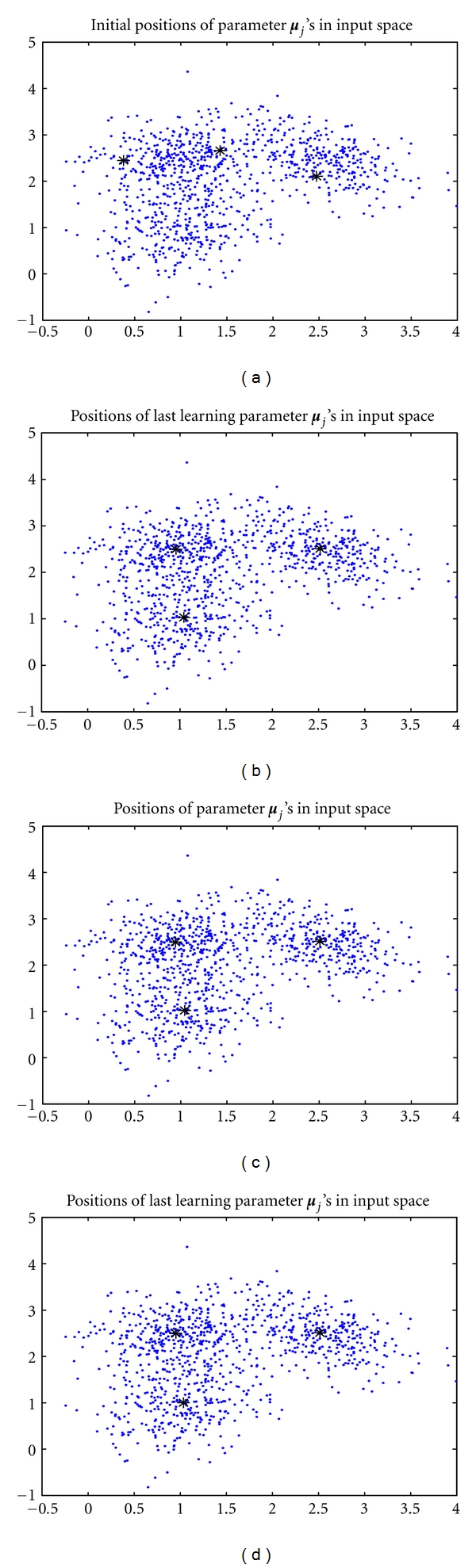
(a) The initial positions of the three seed points and their converged positions learned by (b) EM, (c) adaptive RPEM, and (d) batch RPEM, respectively.

We let *k* = 3, which is equal to the true mixture number k* = 3. The three seed points were randomly allocated in the observation space as shown in Figure 5(a), where the data are considerably overlapped. Moreover, all *α*_*j*_'s and **C**_*j*_'s were initialized at 1/*k* and the identity matrix, respectively.  Figure 5(d) shows the positions of the three converged seed points, which are all stably located at the corresponding cluster centers. For comparison, we also implemented the EM under the same experimental environment.  Figure 5(b) shows that the EM had successfully located the three seed points as well as the batch RPEM.

**Figure 6:**
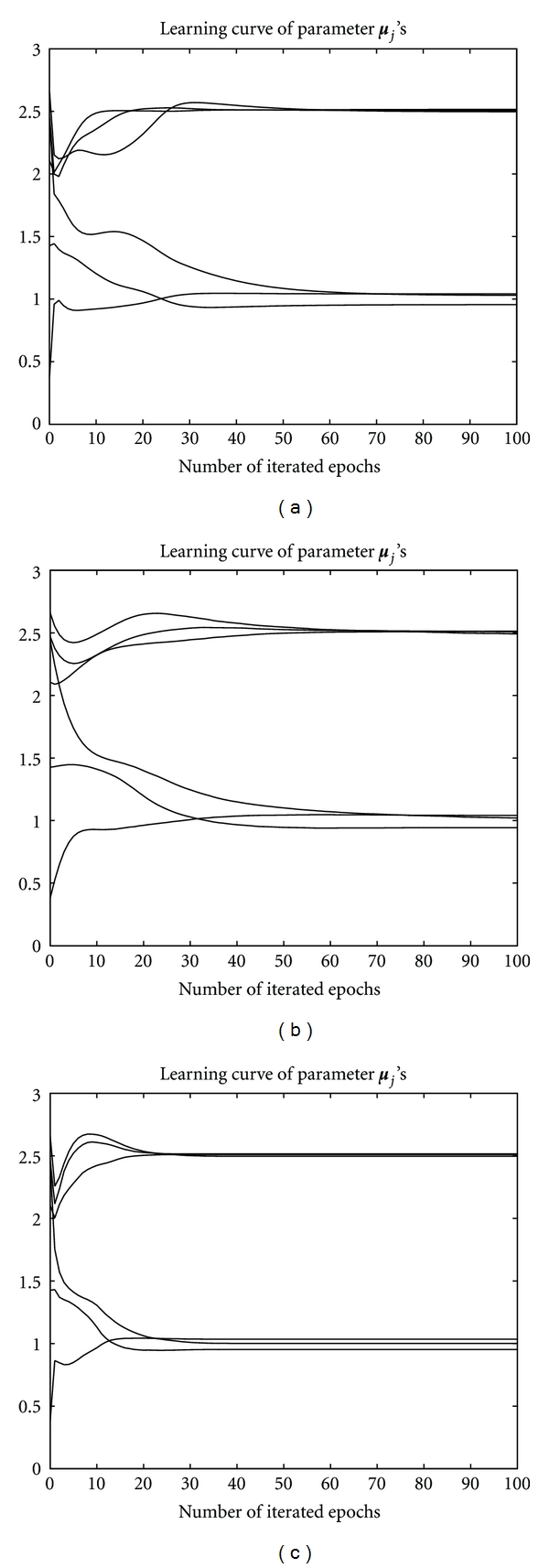
Learning curves of ***μ***_*j*_'s by (a) EM, (b) adaptive RPEM, and (c) batch RPEM, respectively.

**Figure 7:**
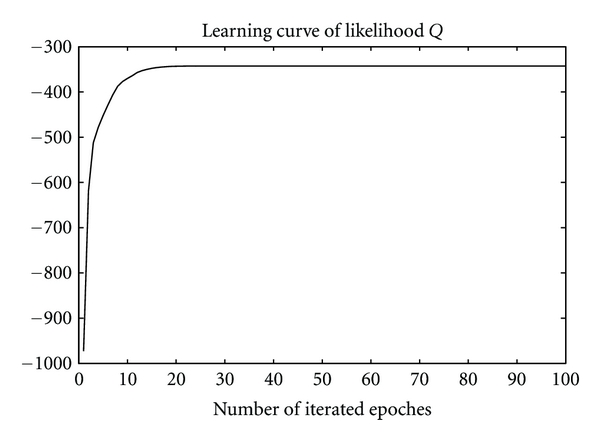
The value of the cost function *Q* over the epochs.

Nevertheless, as shown in Figures 6(c) and 7, the batch RPEM converges at 20 epochs, while the EM needs 60 epochs as shown in Figure 6(a). That is, the convergence speed of the batch RPEM is significantly faster than the EM. This indicates that the intrinsic rival-penalization scheme of the batch RPEM, analogous to the RPCL [11], RPCCL [12], and the adaptive RPEM [14], is able to drive away the rival seed points so that they can be more quickly towards the other cluster centers. As a result, the batch RPEM converges much faster than the EM. Moreover, we also compared it with the adaptive RPEM, in which we set the learning rate *η* = 0.001.  Figure 5(c) shows the convergent results of the seed points. It can be seen that the adaptive RPEM works quite well in this case, but it needs around 70 epochs as shown in Figure 6(b). Actually, the adaptive RPEM can be further speed up if an appropriate learning rate is adopted, which, however, is not a trivial task.

### 5.2. Experiment  2: Batch RPEM on Synthetic Data with *K* > K*

This experiment will investigate the performance of batch RPEM performance as *k* > k*. We generated 1,000 observations from a mixture of five bivariate Gaussian density distributions with the mixing proportions:

(17) $$\begin{array}{c} \alpha_{1}^{*}=0.1,\;\alpha_{2}^{*}=0.2,\;\alpha_{3}^{*}=0.3,\;\alpha_{4}^{*}=0.2,\;\alpha_{5}^{*}=0.2 \end{array}$$

and the true cluster centers:

(18) $$\begin{array}{c} \mu_{1}^{*}={\left[1.0,1.0\right]}^{T},{\;\mu }_{2}^{*}={\left[1.0,2.5\right]}^{T},\;\mu_{3}^{*}={\left[2.5,2.5\right]}^{T},\;\\ \mu_{4}^{*}={\left[2.5,1.0\right]}^{T},\;\mu_{5}^{*}={\left[4.0,2.0\right]}^{T}. \end{array}$$

**Figure 8:**
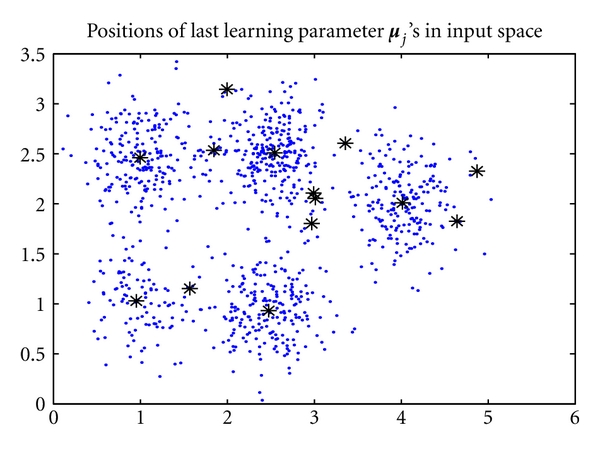
The converged positions of the seed points learned by the batch RPEM.

15 seed points were initialized in the input space arbitrarily. During the learning, the three density components were discarded because their corresponding covariances became singular. As a result, the remaining 12 converged proportions were *α*_1_ = 0.0065, *α*_2_ = 0.0113, *α*_3_ = 0.1929, *α*_4_ = 0.0030, *α*_5_ = 0.0068, *α*_6_ = 0.2013, *α*_7_ = 0.2084, *α*_8_ = 0.0074, *α*_9_ = 0.0083, *α*_10_ = 0.0986, *α*_11_ = 0.2531, and *α*_12_ = 0.0022. It can be seen that the five principal values *α*_3_, *α*_6_,*α*_7_, *α*_10_, and *α*_11_ were estimated well, while the others were learned towards zero. A snapshot of the corresponding ***μ***_*j*_'s were ***μ***_3  _ = [4.0348,2.0075]^*T*^, ***μ***_6_ = [0.9990,2.4571]^*T*^, ***μ***_7_ = [2.4725,0.9220]^*T*^, ***μ***_10_ = [0.9553,1.0277]^*T*^, and ***μ***_11_ = [2.5189,2.5199]^*T*^. As shown in Figure 8, these five seed points have successfully allocated in the cluster centers, meanwhile the batch RPEM drove the redundant seed points to the boundaries of the clusters.

### 5.3. Experiment  3: Batch RPEM on Color Image Segmentation

**Figure 9:**
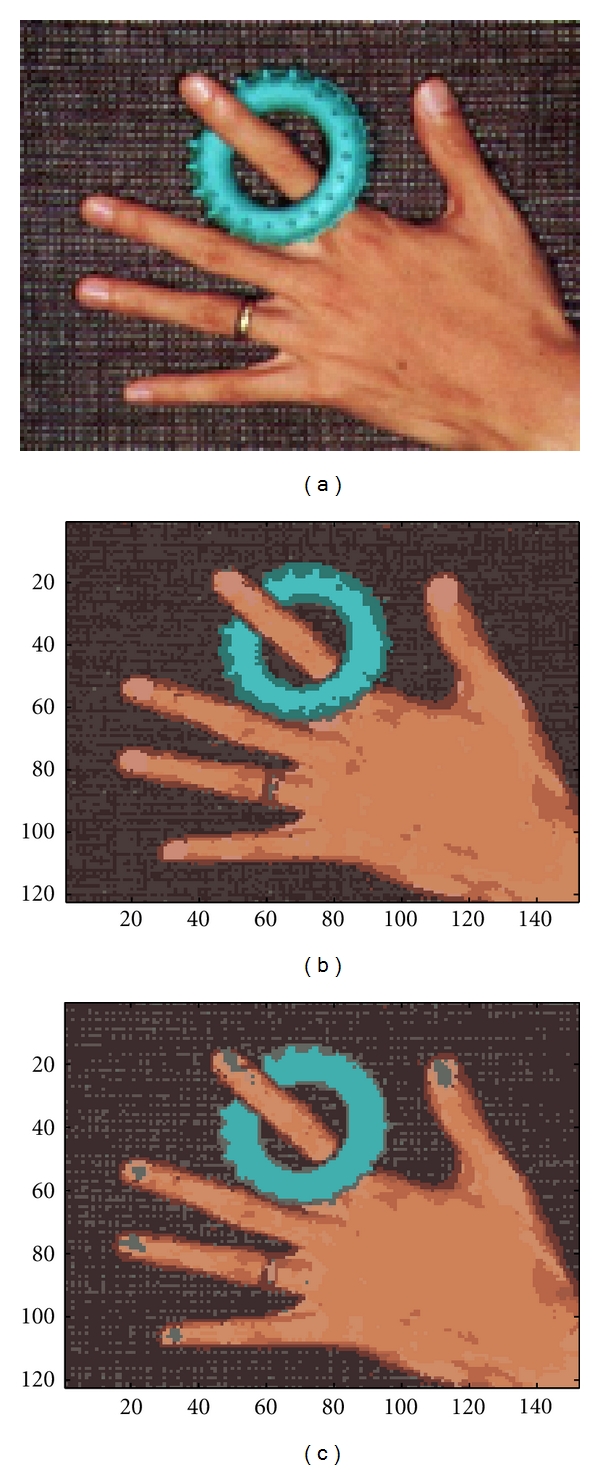
Segmentation of the hand image: (a) original image, (b) the result given by the EM, and (c) the result given by the batch RPEM.

**Figure 10:**
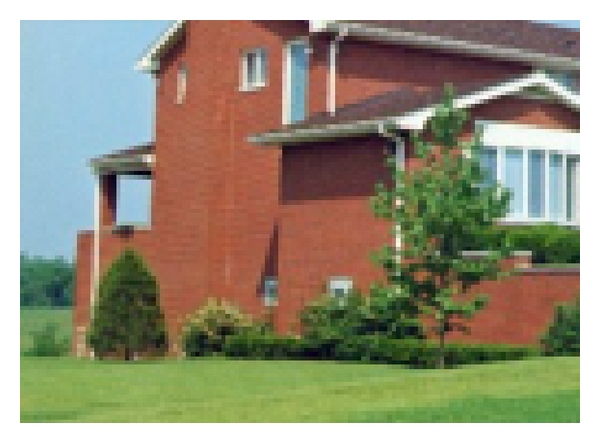
The original house image.

This experiment further investigated the batch RPEM algorithm on color image segmentation in comparison to the EM algorithm. We implemented the image segmentation in the red-green-blue (RGB) color space model that represents each pixel in an image by a three-color vector. We conducted color image segmentation on a 122 × 152 hand image and a 96 × 128 house image as shown in Figures 9(a) and 10, respectively. For the former, we initially assigned 10 seed points randomly. After the convergence of the algorithms' performance, a snapshot of their segmentation results is shown in Figures 9(b) and 9(c). It can be seen that the blue tiny swim ring-shaped region after segmentation process by the batch RPEM is smoother than the EM. Further, the tiny nail regions have been partitioned by the batch RPEM but the EM is not. In other words, the batch RPEM algorithm performs better than the EM algorithm.

**Figure 11:**
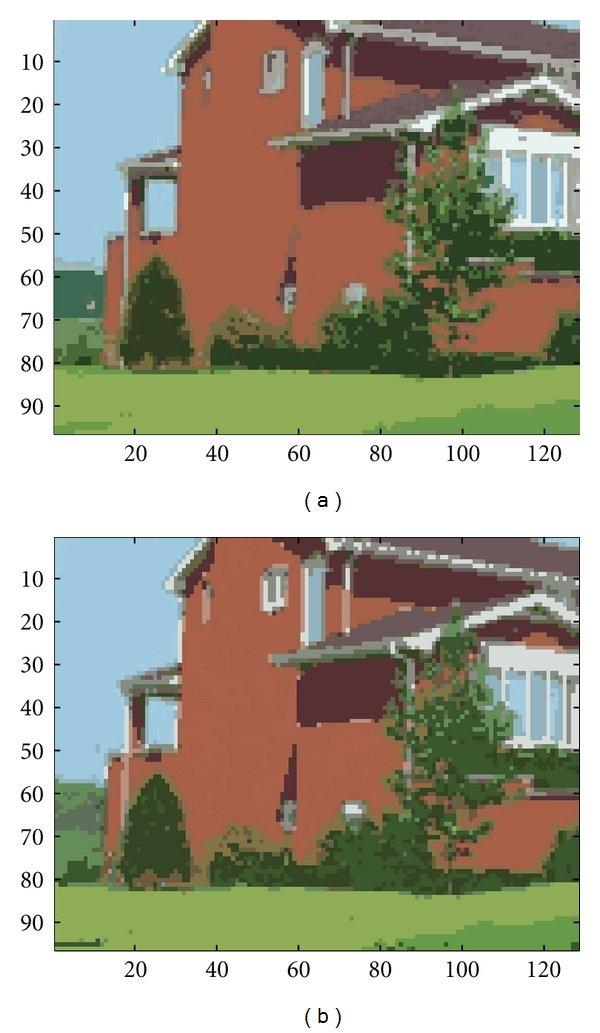
Segmentation of the house image by (a) EM; (b) batch RPEM.

For the house image, we initially assigned the seed points to be 80. A snapshot of the converged segmentation results of the EM and the batch RPEM is shown in Figure 11. It can be seen that the texture on the red wall and the green lawn has no longer maintained after the segmentation process both by the EM and the RPEM. However, the small white regions of windows on red wall were disappeared by the EM as well as the triangle shadow area on the wall. In contrast, the batch RPEM algorithm partitioned these regions well as shown in Figure 11(b). Actually, the batch RPEM has drove out the redundant seed points far away and maintained some principal components correctly, which therefore leads to a better performance in color image segmentation.

## 6. Conclusion 

In this paper, we have developed a batch RPEM algorithm based on MWL learning framework for Gaussian mixture clustering. Compared to the adaptive RPEM, this new one need not select the value of learning rate. As a result, it can learn faster in general and still preserve the capability of automatic model selection analogous to the adaptive one. We have evaluated the proposed batch RPEM algorithm on both synthetic data and color image segmentation. The numerical results have shown the efficacy of the proposed algorithm.

## Figures and Tables

**Algorithm 1:**
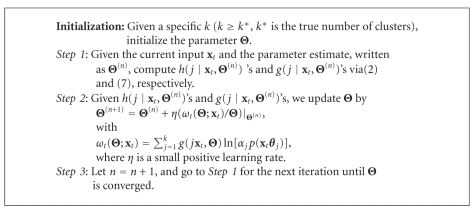
Adaptive RPEM algorithm.

**Algorithm 2:**
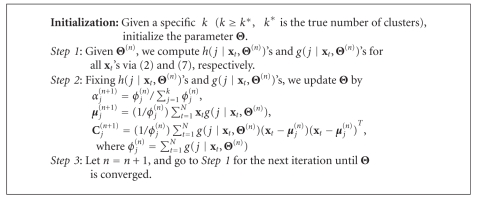
Batch RPEM algorithm.

**Table 1:** Performance of the Batch RPEM over the Parameter *ɛ*, where “G” stands for a good model selection capability of the algorithm, while “P” represents a poor model selection capability.

*ɛ*	k* = 3, *k* = 3		k* = 3, *k* = 8		k* = 3, *k* = 20	
	Data set 1	Data set 2	Data set 1	Data set 2	Data set 1	Data set 2
−0.9	G	G	G	G	G	G
−0.8	G	G	G	G	G	G
−0.7	G	G	G	G	G	G
−0.6	G	G	G	G	G	G
−0.5	G	P	G	P	G	G
−0.4	G	P	G	P	P	P
−0.3	G	P	G	P	P	P
−0.2	G	P	G	P	P	P
−0.1	G	P	G	P	P	P

## References

[1] Fayyad U, Piatetsky-Shpiro G, Smyth P, Uthurusamy R (1996). *Advances in Knowledge Discovery and Data Mining*.

[2] Fritzke B (1997). The LBG-U method for vector quantization—an improvement over LBG inspired from neural networks. *Neural Processing Letters*.

[3] Linde Y, Buzo A, Gray R (1980). An algorithm for vector quantizer design. *IEEE Transactions on Communications*.

[4] Chen SY, Tong HY, Wang ZJ, Liu S, Li M, Zhang BW (2011). Improved generalized belief propagation for vision processing. *Mathematical Problems in Engineering*.

[5] Chen SY, Tong HY, Cattani C (2012). Markov models for image labeling. *Mathematical Problems in Engineering*.

[6] Lim YW, Lee SU (1990). On the color image segmentation algorithm based on the thresholding and the fuzzy C-means techniques. *Pattern Recognition*.

[7] Uchiyama T, Arib MA (1994). Color image segmentation using competitive learning. *IEEE Transactions on Pattern Analysis and Machine Intelligence*.

[8] Jolion JM, Meer P, Bataouche S (1991). Robust clustering with applications in computer vision. *IEEE Transactions on Pattern Analysis and Machine Intelligence*.

[9] Dempster A, Laird N, Rubin D (1977). Maximum likelihood from incomplete data via the EM algorithm. *Journal of the Royal Statistical Society B*.

[10] Jeff BilmesA (1998). *A Gentle Tutorial of the EM Algorithm and Its Application to Parameter Estimation for Gausssian Mixture and Hidden Markov Models*.

[11] Xu L, Krzyzak A, Oja E (1993). Rival penalized competive learning for clustering analysis, RBF Net, and curve detection. *IEEE Transactions on Neural Networks*.

[12] Cheung YM Rival penalized controlled competitive learning for data clustering with unknown cluster number.

[13] Cheung YM A rival penalized EM algorithm towards maximizing weighted likelihood for density mixture clustering with automatic model selection.

[14] Cheung YM (2005). Maximum weighted likelihood via rival penalized EM for density mixture clustering with automatic model selection. *IEEE Transactions on Knowledge and Data Engineering*.

[15] Zhao XM, Cheung YM, Chen L, Aihara K A new technique for adjusting the learning rate of RPEM algorithm automatically.

